# Efficacy of osimertinib in patients with *EGFR* ‐mutation positive non‐small cell lung cancer with malignant pleural effusion

**DOI:** 10.1111/1759-7714.15210

**Published:** 2024-01-16

**Authors:** Ayu Kiritani, Yoshiaki Amino, Ken Uchibori, Takahiro Akita, Yuhei Harutani, Shinsuke Ogusu, Ryosuke Tsugitomi, Ryo Manabe, Ryo Ariyasu, Satoru Kitazono, Noriko Yanagitani, Makoto Nishio

**Affiliations:** ^1^ Department of Respiratory Medicine Jikei University School of Medicine Minato Japan; ^2^ Department of Thoracic Medical Oncology Cancer Institute Hospital of Japanese Foundation for Cancer Research Koto Japan; ^3^ Department of Respiratory Medicine Hachinohe City Hospital Hachinohe Japan; ^4^ Department of Internal Medicine III Wakayama Medical University Wakayama Japan; ^5^ Division of Hematology, Respiratory Medicine and Oncology, Department of Internal Medicine Faculty of Medicine, Saga University Saga Japan; ^6^ Division of Allergology and Respiratory Medicine, Department of Internal Medicine Showa University School of Medicine Shinagawa Japan

**Keywords:** EGFR, malignant pleural effusion, non‐small cell lung cancer, osimertinib

## Abstract

**Background:**

As an epidermal growth factor receptor‐tyrosine kinase inhibitor (EGFR‐TKI), osimertinib has emerged as a standard *EGFR*‐mutation positive treatment for non‐small cell lung cancer (NSCLC). However, the efficacy of osimertinib for malignant pleural effusion (MPE) remains understudied. This study aimed to evaluate the impact of osimertinib on time to treatment failure (TTF) and overall survival (OS) in patients with *EGFR*‐mutation positive NSCLC, comparing those with and without MPE.

**Methods:**

This retrospective analysis included patients with advanced or recurrent NSCLC treated with osimertinib at our hospital between April 2016 and June 2021. TTF was defined as the duration from osimertinib initiation to discontinuation, and OS as the duration until death, irrespective of the reason.

**Results:**

Among 229 patients receiving osimertinib, 84 had MPE before administration, 39 acquired EGFR exon20 T790M mutation following previous EGFR‐TKI therapy, and 45 were EGFR‐TKI‐naive. Among EGFR‐TKI‐naive patients, median TTF was 14.8 and 19.8 months for those with and without MPE, respectively (hazard ratio [HR] 1.40; 95% confidence interval [CI]: 0.90–2.18; *p* = 0.12). Median OS was 32.0 and 42.0 months for patients with and without MPE, respectively (HR 1.43; 95% CI: 0.86–2.38; *p* = 0.16). Among patients with T790M mutation, median TTF was 12.3 and 13.1 months for patients with and without MPE, respectively (HR 1.03; 95% CI: 0.69–1.55; *p* = 0.88). Median OS for patients with and without MPE was 23.2 and 24.7 months, respectively (HR 1.09; 95% CI: 0.72–1.67; *p* = 0.68).

**Conclusion:**

Among patients with *EGFR*‐mutation positive NSCLC, the evidence of MPE has little effect on survival with osimertinib.

## Graphical abstract

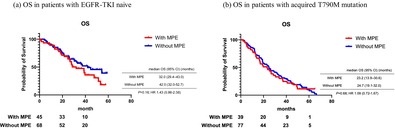

## INTRODUCTION

Epidermal growth factor receptor tyrosine kinase inhibitors (EGFR‐TKIs) play a crucial role in the treatment of *EGFR*‐mutation positive non‐small cell lung cancer (NSCLC).^1^ Among these drugs, osimertinib is the most used and considered the standard of care; it is also regarded as a second‐line treatment after prior EGFR‐TKIs administration. The FLAURA trial reported that osimertinib significantly improved progression‐free survival (PFS) and overall survival (OS) compared with gefitinib or erlotinib in untreated patients.^2^ Additionally, the AURA3 trial demonstrated that osimertinib significantly prolonged PFS in patients with acquired EGFR T790M secondary mutation after prior EGFR‐TKIs compared to chemotherapy.^3^ However, the efficacy of EGFR‐TKIs may be influenced by various patient‐related clinical characteristics including central nervous system metastases,^4^ smoking status,^5^ and the presence of squamous cell carcinoma,^6^ which may impact treatment response. Malignant pleural effusion (MPE), recognized as a poor prognosis factor, has been associated with suboptimal response to first‐ and second‐generation EGFR‐TKIs in previous studies.^7^, ^8^ Notably, there is a lack of clinical data directly comparing the efficacy of osimertinib between patients with and without pleural effusion.^9^, ^10^, ^11^, ^12^ Therefore, this study aimed to evaluate the efficacy of osimertinib and the prognosis of patients treated with osimertinib in patients with *EGFR*‐mutation positive NSCLC, accounting for the presence of MPE.

## METHODS

### Patient cohort

We retrospectively reviewed the medical records of patients with advanced or recurrent *EGFR*‐mutation positive NSCLC treated with osimertinib at our hospital between April 2016 and June 2021. This study investigated patients who matched the following criteria: (1) had pathologically confirmed NSCLC, (2) with an *EGFR* mutation confirmed in tumor tissue or cytology specimens. A cobas *EGFR* mutation test version 2 or Oncomine Dx target test was used to evaluate and identify *EGFR* mutations, and (3) had received osimertinib treatment regardless of whether previously treated.

### Data collection

Clinical data including age, sex, smoking status, Eastern Cooperative Oncology Group (ECOG) performance status (PS), tumor histology type, *EGFR* mutation status, clinical stage at diagnosis, metastatic sites, and history of prior treatment were collected from electronic medical records and databases. Treatment efficacy was evaluated by computed tomography (CT) scanning; CT scanning was performed at least every 3 months. Clinical response was evaluated according to the Response Evaluation Criteria in Solid Tumors, version 1.1 (RECIST version 1.1). Time to treatment failure (TTF) was defined as the interval from the initiation of osimertinib to treatment discontinuation for any reason. Overall survival (OS) was calculated from the initiation of osimertinib to the date of death or August 2023, defined as the cutoff time. Pleural effusion with a thickness of 10 mm or more depicted by chest CT scanning at the initiation of osimertinib was described as a case with “MPE”.^13^ Adverse events were evaluated according to the National Cancer Institute Common Terminology Criteria for Adverse Events version 5.0.

### Statistical analysis

TTF and OS were estimated using the Kaplan–Meier method, and the differences in survival times between each group were assessed with the log‐rank test. The Cox proportional hazard model was used for univariate analysis. All statistical analyses were conducted using GraphPad Prism version 9.0 (GraphPad Software) or JMP version 14.2 software (SAS Institute Inc.). A *p*‐value <0.05 was considered statistically significant, and *p* < 0.10 was considered marginally different. In multiple analysis, factors with *p* < 0.1 in the univariate analysis were included.

### Ethical considerations

The study was conducted in accordance with the principles of the Declaration of Helsinki and was approved by the Institutional Review Board of the participating hospitals (approval number: C‐T2022‐0002).

## RESULTS

### Patient characteristics

**FIGURE 1:**
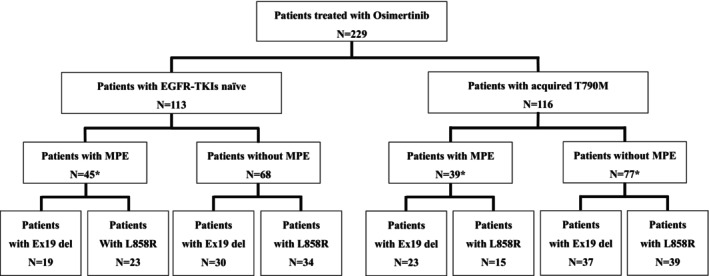
Patient flow chart. Exon 19 deletion, EGFR exon 19 deletion; L858R, EGFR exon21 L858R; MPE, malignant pleural effusion. *The number of patients included those with *EGFR* minor mutation.

Figure 1 illustrates the patient flow chart of this study. A total of 229 patients were treated with osimertinib; 113 patients were EGFR‐TKI treatment‐naïve, and 116 patients had acquired *EGFR* exon 20 T790M mutation after prior EGFR‐TKI treatment. Among the total patient cohort (*n* = 229), MPE was identified in 84 patients (36.6%). Baseline characteristics of patients with/without MPE are shown in Table 1. Patients with MPE exhibited a higher incidence of poor PS and stage IV than those without MPE (*p* = 0.006 and *p* = 0.001, respectively). Brain metastases (37.9%) were more prevalent in patients with MPE, although this difference was not significant (*p* = 0.07). There was no difference in the rate of EGFR subtypes between patients with and without MPE among EGFR‐TKI‐naïve patients and those with acquired T790M. Characteristics of the 113 EGFR‐TKIs naïve patients and the 116 patients with developed T790M are shown in Table 1. Among the EGFR‐TKI‐naïve patients, 39.8% exhibited MPE, while 31.0% of patients with acquired T790M exhibited MPE. There were no significant differences in the rate of patients with MPE between TKI naïve patients and T790M acquired patients. No significant differences were detected between patients with MPE and those without MPE. In patients with developed T790M, those with MPE were older, with a higher proportion of females than the proportion in those without MPE (*p* = 0.02 and *p* < 0.001, respectively). In EGFR‐TKI‐naïve patients, no differences were observed by age or gender. Overall, poorer PS was depicted in patients with MPE (*p* = 0.07). However, no correlation was observed between MPE and PS in EGFR‐TKI‐naïve patients and those with acquired T790M.

**TABLE 1:** Patient characteristics.

	All patients		*p*‐value	EGFR‐TKI naïve		*p*‐value	Acquired T790M		*p*‐value
	With MPE	Without MPE		With MPE	Without MPE		With MPE	Without MPE	
	(*N* = 84)	(*N* = 145)		(*N* = 45)	(*N* = 68)		(*N* = 39)	(*N* = 77)	
Age, median (range)	68.0 (37–68)	65.0 (41–84)	0.37	71.0 (37–86)	67.0 (40–86)	0.36	67.0 (41–84)	63.0 (37–87)	0.02
Sex, *N* (%)									
Male	27 (32.1)	50 (34.5)	0.71	12 (26.7)	23 (33.8)	0.55	24 (61.5)	13 (16.9)	<0.001
Female	57 (67.9)	95 (65.5)		33 (73.3)	45 (66.2)		15 (38.5)	64 (83.1)	
ECOG PS, *N* (%)									
0–1	71 (84.5)	138 (95.2)	0.006	38 (84.4)	64 (94.1)	0.17	33 (84.6)	74 (96.1)	0.07
≥2	13 (15.5)	7 (4.8)		7 (15.6)	4 (5.9)		6 (15.4)	3 (3.9)	
Smoking status, *N* (%)									
Never	54 (65.1)	90 (62.1)	0.65	29 (64.4)	41 (60.3)	0.81	26 (66.7)	49 (63.6)	0.91
Current or ex	30 (34.9)	55 (37.9)		16 (35.5)	27 (39.7)		13 (33.3)	28 (36.4)	
Stage, *N* (%)									
III	10 (11.9)	2 (1.4)	0.001	0	6 (8.8)	0.1	1 (2.6)	5 (6.5)	0.65
IV or recurrence	74 (88.1)	143 (98.6)		45 (100)	62 (91.2)		38 (97.4)	72 (93.5)	
Metastatic state, *N* (%)									
Brain	22 (26.2)	55 (37.9)	0.07	11 (24.4)	20 (29.4)	0.72	11 (28.2)	35 (45.5)	0.11
Liver	12 (14.3)	21 (14.5)	0.97	7 (15.6)	7 (10.3)	0.59	5 (12.8)	14 (18.2)	0.63
Bone	28 (33.3)	62 (42.3)	0.16	12 (26.7)	28 (41.2)	0.16	16 (41.0)	34 (44.2)	0.9
Lung	41 (48.1)	66 (45.5)	0.63	16 (35.6)	16 (23.5)	0.24	25 (64.1)	50 (64.9)	0.47
Mutation type, *N* (%)									
Exon19 deletion	42 (50.0)	65 (44.8)	0.69	19 (42.2)	30 (44.1)	1.00	23 (58.9)	37 (48.1)	0.36
Exon21 L858R	38 (45.2)	75 (51.7)		23 (51.1)	34 (50.0)		15 (38.5)	39 (50.6)	
Others	4 (4.8)	3 (3.5)		3 (3.6)	4 (5.9)		1 (2.6)	1 (1.3)	

Abbreviations: ECOG PS, Eastern Cooperative Oncology Group performance status; EGFR‐TKI, epidermal growth factor receptor‐tyrosine kinase inhibitor; MPE, malignant pleural effusion.

### Efficacy

**FIGURE 2:**
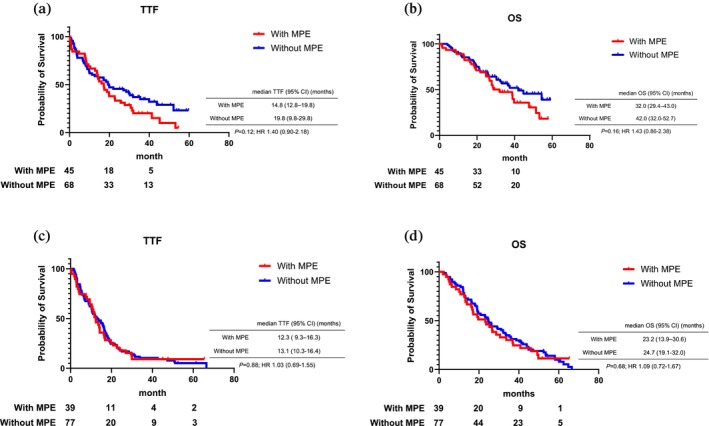
Time to treatment failure and survival of patients with and without MPE treated osimertinib overall. MPE, malignant pleural effusion; OS, overall survival; TTF, time to treatment failure. (a) TTF in patients with EGFR‐TKI naïve. (b) OS in patients with EGFR‐TKI naïve. (c) TTF in patients with acquired T790M mutation. (d) OS in patients with acquired T790M mutation.

The median follow‐up period from the administration of osimertinib was 29.0 months in EGFR‐TKI‐naïve patients and 24.6 months in those with acquired T790M. In EGFR‐TKI naïve patients and patients with acquired T790M mutation, the overall response rate (ORR) was higher in groups without MPE than in groups with MPE (40.0% vs. 50.0%, 48.7% vs. 59.7%: Table S2). Kaplan–Meier TTF and OS curves are shown in Figure 2. The median TTF of osimertinib in EGFR‐TKI‐naïve patients with and without MPE was 14.8 and 19.8 months, respectively, with a nonsignificant difference (Figure 2a). The median TTF of osimertinib in patients with acquired T790M with and without MPE was 12.3 and 13.1 months, which was not significant (Figure 2c).

The median OS in EGFR‐TKI‐naïve patients with and without MPE were 32.0 and 42.0 months, respectively (*p* = 0.16, Figure 2b). The median OS in previously treated patients with and without MPE was 23.2 and 24.7 months, respectively, and was not significant (*p* = 0.68, Figure 2d).

Univariate analysis results of TTF and OS in EGFR‐TKI‐naive patients and those with acquired T790M are shown in Table 2. PS affected TTF and OS in EGFR‐TKI‐naïve patients and those with acquired T790M. In EGFR‐TKI‐naïve patients, brain and liver metastases were associated with TTF, whereas brain metastases and MPE affected OS. In patients with acquired T790M, EGFR subtypes affected TTF and OS, whereas liver metastases were correlated with OS.

**TABLE 2:** Univariate analyses of TTF and OS in patients with treatment naïve and acquired T790M mutation.

	EGFR‐TKI naïve				Acquired T790M			
	TTF		OS		TTF		OS	
	HR (95% CI)	*p*‐value	HR (95% CI)	*p*‐value	HR (95% CI)	*p*‐value	HR (95% CI)	*p*‐value
Age								
<75/≥75	1.35 (0.84–2.09)	0.38	0.97 (0.56–1.68)	0.92	1.02 (0.65–1.58)	0.92	1.00 (0.63–1.58)	1.00
Sex								
Female/male	0.95 (0.59–1.54)	0.84	0.76 (0.42–1.31)	0.30	1.00 (0.68–1.47)	0.99	1.00 (0.68–1.49)	0.99
ECOG PS								
0–1/≥2	0.34 (0.11–1.03)	0.001	0.29 (0.10–0.84)	<0.0001	0.46 (0.17–1.20)	0.02	0.34 (0.11–1.02)	0.0009
Smoking status								
Never/current or ex	0.92 (0.59–1.45)	0.22	0.64 (0.38–1.08)	0.07	1.02 (0.69–1.50)	0.93	0.95 (0.64–1.42)	0.81
*EGFR* mutation								
Exon 19 deletion/Ex 21 L858R	0.92 (0.53–1.30)	0.41	1.21 (0.72–2.03)	0.46	0.71 (0.47–1.02)	0.05	0.68 (0.46–1.01)	0.05
Metastatic state								
MPE	1.40 (0.90–2.18)	0.12	1.43 (0.86–2.38)	0.16	1.03 (0.69–1.55)	0.88	1.09 (0.72–1.67)	0.68
Brain metastases	1.82 (1.09–3.03)	0.01	2.36 (1.28–4.34)	0.0007	1.18 (0.80–1.74)	0.38	0.84 (0.57–1.25)	0.37
Liver metastases	1.80 (0.83–3.87)	0.06	1.57 (0.71–3.49)	0.19	1.28 (0.74–2.20)	0.32	1.53 (0.86–2.71)	0.09
Bone metastases	1.03 (0.66–1.61)	0.91	1.11 (0.66–1.88)	0.69	1.11 (0.76–1.62)	0.60	1.22 (0.82–1.80)	0.31
Lung metastases	1.34 (0.82–2.20)	0.21	1.10 (0.64–1.91)	0.72	1.06 (0.17–1.58)	0.76	1.06 (0.71–1.58)	0.76

Abbreviations: CI, confidence interval; ECOG PS, Eastern Cooperative Oncology Group performance status; HR, hazard ratio; MPE, malignant pleural effusion; OS, overall survival; TTF, time to treatment failure.

Forward and back‐forward stepwise multiple regression analyses depicted that brain metastases and liver metastases were significantly and independently correlated with poor OS in EGFR‐TKI‐naive patients. In patients with acquired T790M, poor PS correlated with poor OS. In multivariate analysis, MPE was not identified as a factor affecting TTF or OS.

**FIGURE 3:**
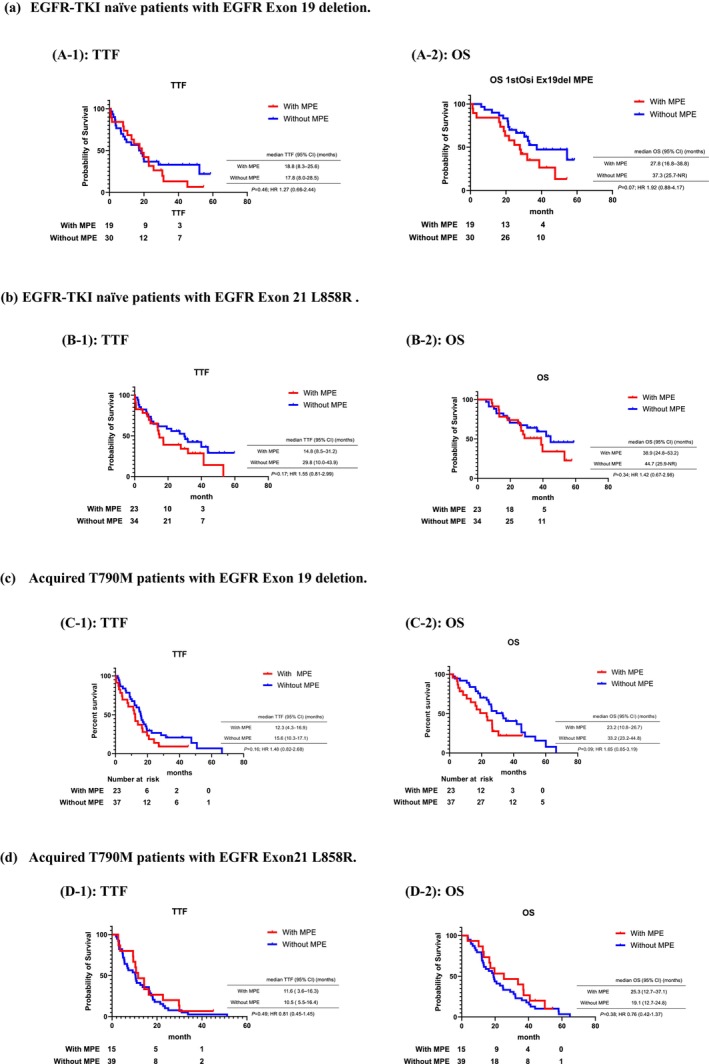
Time to treatment failure and survival of patients with and without MPE treated osimertinib in subtypes of *EGFR* mutations. MPE, malignant pleural effusion; OS, overall survival; TTF, time to treatment failure. (a) EGFR‐TKI naïve patients with EGFR exon 19 deletion. (b) EGFR‐TKI naïve patients with EGFR exon 21 L858R. (c) Acquired T790M patients with EGFR exon 19 deletion. (d) Acquired T790M patients with EGFR exon 21 L858R.

There may be some differences in the efficacy of osimertinib in TTF and OS depending on the subtype of *EGFR* mutation; however, these differences were not significant (Figure 3). There was no significant difference in TTF or OS between patients with and without MPE by *EGFR* mutation subtype. However, for OS of patients who are EGFR‐TKI naïve and with acquired T790M mutation with EGFR exon 19 deletion, there was a slightly larger difference between those with and without MPE (*p* = 0.19, *p* = 0.09, respectively). Additionally, there was a slightly larger difference in TTF of patients with acquired T790M mutation with EGFR exon19 deletion.

In 45 EGFR‐TKI naïve patients with MPE, 19 (42.2%) patients required thoracentesis and three (6.7%) patients required pleural adhesions before administration of osimertinib. In 39 patients with acquired T790M mutation and MPE, 25 patients (64.1%) required thoracentesis and four patients (10.2%) required pleural adhesions before administration of osimertinib. The number of cases with increased MPE after treatment initiation was 12 cases in EGFR‐TKI naïve patients with MPW and eight cases in patients with acquired T790M mutation and MPE. Most of the cases were treated with thoracentesis only, and pleural adhesions were performed in only a few cases.

### Safety analysis

As shown in Table S2, adverse events were observed in 94 (83.2%) EGFR‐TKI naïve patients, and 95 (81.9%) patients with acquired T790M mutation experienced at least one drug‐related adverse event. In EGFR‐TKI naïve patients, 11 (9.7%) required dose reduction of osimertinib, and 12 (10.6%) experienced treatment discontinuation. In patients with acquired T790M mutation, 12 (10.3%) needed dose reduction of osimertinib, and eight (6.9%) experienced treatment discontinuation. The most common adverse event was rash in EGFR‐TKI naïve patients and patients with acquired T790M mutations. No treatment‐related deaths occurred.

## DISCUSSION

In this study, we analyzed TTF and OS among patients with *EGFR*‐mutation positive NSCLC treated with osimertinib depending on the presence or absence of MPE. Primarily, we evaluated the difference in EGFR‐TKI‐naïve patients and those with acquired T790M. Our results showed that, particularly among EGFR‐TKI‐naïve patients with and without MPE, median TTF was 14.8 and 19.8 months, respectively, and median OS was 32.0 and 42.0 months, respectively. The median TTF and OS of patients without MPE were similar to the median PFS (18.9 months) and OS (38.6 months) reported in the FLAURA trial.^14^ Although the median TTF and OS of EGFR‐TKI‐naive patients with MPE were shorter than those found in the FLAURA trial, our result was not significantly different between patients with MPE and those without MPE. In patients with acquired T790M with and without MPE, the median TTF was 12.3 and 13.1 months, respectively, and the median OS was 23.2 and 24.7 months, respectively. The median TTF and OS of patients with acquired T790M mutation were comparable to the median PFS (10.1 months) and OS (26.8 months) reported in the AURA3 trial.^3^ In these patients with MPE, although the median TTF and OS were shorter than the results of the AURA3 trial, there was no significant difference. In our evaluation of the association between the efficacy of osimertinib and metastatic lesions, brain metastasis emerged as an independent factor in EGFR‐TKI‐naïve patients. However, in patients with acquired T790M, poor PS was the only associated factor, while no associations were depicted with metastatic sites. The univariate and multivariate analyses we conducted also did not show an association between MPE and the efficacy of osimertinib.

The efficacy of EGFR‐TKI in patients with MPE has been previously reported,^9^, ^10^, ^11^, ^12^ and previous studies have indicated a significant association between *EGFR* mutation and MPE.^15^, ^16^ Moreover, first‐ and second‐generation EGFR‐TKIs have demonstrated poor effectiveness in cases of pleural effusion.^7^, ^8^ The efficacy of osimertinib for pleural effusion was previously evaluated in some studies^9^, ^10^, ^11^, ^12^ encompassing previously treated patients with the acquired T790M mutation. Masuhiro et al.^9^ and Kawamura et al.^10^ also evaluated the effect of osimertinib for MPE, in 23 and 90 patients with acquired T790M, respectively. Both studies concluded that MPE was associated with poor prognosis. By contrast, a retrospective analysis^12^ of 3578 patients previously treated and with acquired T790M mutation shed light on the effectiveness of osimertinib regardless of MPE status. Furthermore, Nokihara et al.^11^ evaluated the efficacy of osimertinib for MPE within treated patients carrying the T790M mutation and untreated patients. They reported that in treated patients with T790M (*n* = 30), PFS and OS were significantly better in patients without than in those with pleural effusion (median PFS, 16.8 months vs. 8.3 months, *p* = 0.003; median OS, 44.9 months vs. 14.2 months, *p* = 0.007). Among untreated (T790M) negative patients (*n* = 33), there were no differences in PFS or OS between those with MPE and those without (median PFS, 19.8 months vs. 19.8 months, *p* = 0.693; median OS, NR vs. NR, *p* = 0.712). Another retrospective multicenter cohort study encompassing 538 patients with an *EGFR* mutation who received osimertinib as initial therapy^17^ reported that PFS for malignant effusion, which included pericardial effusion and pleural/ascites effusion, was low (HR 1.51; 95% CI: 1.11–2.04). In our study, evaluating the effects of osimertinib among untreated and previously treated patients, TTF and OS did not exhibit significant differences between patients with and without MPE. The reason for the differences between our results and those of previous studies is as follows. First, in our analysis including all groups, while the median TTF and OS were shorter than without MPE, there were no significant differences owing to the small number of patients. Second, it may have influenced our results to use TTF instead of PFS, as it is difficult to evaluate the progression of pleural effusion, and TTF may be more reflective of real‐world clinical effects than PFS.

The difference in response to osimertinib between untreated and previously treated patients may be related to different patterns of resistance development against osimertinib between EGFR‐TKI‐naive and T790M‐positive patients. The different resistance mechanisms between the AURA3 and FLAURA studies^18^, ^19^ may explain these differences, mainly the higher frequency of C797S mutation in AURA3 than in FLAURA (14% vs. 6%). Previous reports have suggested that EGFR subtypes influenced EGFR‐TKI response.^20^, ^21^ In a subset analysis of FLAURA,^14^ PFS and OS of osimertinib differed between EGFR exon 19 deletion and exon 21 L858R. Our study investigated the difference in efficacy of osimertinib for MPE in EGFR exon 19 deletion and exon 21 L858R. Although no significant differences were found among patients with exon 19 deletion, there was a slightly larger difference in OS between patients with and without MPE, with larger differences in the TTF of patients with acquired T790M. There may also be differences in the effect of osimertinib on MPE due to *EGFR* mutation subtype and the presence of T790M mutation.

This study had some limitations. A collection bias may have been present owing to the retrospective study design; the sample size was small as participants were recruited from a single institution. In addition, the mechanism of resistance in MPE was not examined.

In conclusion, we found no significant differences between TTF and OS observed in the presence or absence of MPE in patients with *EGFR*‐mutation positive NSCLC treated with osimertinib. However, *EGFR* mutation subtypes and T790M mutation status may make a difference in the effect of osimertinib on pleural effusion.

## AUTHOR CONTRIBUTIONS

All authors had full access to the data in the study and take responsibility for the integrity of the data and the accuracy of the data analysis. Conceptualization, Ayu Kiritani, Yoshiaki Amino, Ken Uchibori, and Makoto Nishio; Investigation, Ayu Kiritani, and Yoshiaki Amino; Formal analysis, Ayu Kiritani, and Yoshiaki Amino; Resources, Ayu Kiritani, Yoshiaki Amino and Makoto Nishio; Writing‐original draft, Ayu Kiritani, Yoshiaki Amino and Makoto Nishio; Writing‐review and editing, Ayu Kiritani, Yoshiaki Amino, Ken Uchibori, Yuhei Harutani, Shinsuke Ogusu, Ryosuke Tsugitomi, Ryo Manabe, Ryo Ariyasu, Satoru Kitazono, Noriko Yanagitani, and Makoto Nishio; Visualization, Ayu Kiritani and Yoshiaki Amino; Supervision, Makoto Nishio.

## CONFLICT OF INTEREST STATEMENT

Dr Nishio received honoraria from Novartis, ONO Pharmaceutical, Chugai Pharmaceutical, Bristol‐Myers Squibb, TAIHO Pharmaceutical, Eli Lilly, Pfizer, Astellas Pharma, Daiichi Sankyo, MSD, Abbvie, Takeda Pharmaceutical, Boehringer Ingelheim, Nippon Kayaku, Merck, Janssen and AstraZeneca. Dr Yanagitani received honoraria from Chugai Pharmaceutical, ONO Pharmaceutical, Bristol‐Myers Squibb, AstraZeneca, Eli Lilly, Pfizer, and Takeda Pharmaceutical. Dr Kitazono received honoraria from AstraZeneca, Chugai Pharmaceutical, ONO Pharmaceutical, and Pfizer. Dr Uchibori received honoraria from Astra Zeneca, Amgen, Chugai Pharmaceutical, Takeda Pharmaceutical, ONO Pharmaceutical, Novartis, Eli‐Lilly, Thermo Fisher, Bristol Myers Squib, Merck and Daiichi‐Sankyo. Dr Ariyasu received honoraria from Astra Zeneca, Chugai, and Bristol Myers Squib. All other authors have stated that they have no conflicts of interest.

## Supporting information


**Supplementary Table S1.** Efficacy of osimertinib.
Supplement Table S2. Adverse events.
