# Supplementary material for: Efficacy of osimertinib in patients with EGFR ‐mutation positive non‐small cell lung cancer with malignant pleural effusion
Source: Thorac Cancer. 2024 Jan 16;15(5):402–9. doi: 10.1111/1759-7714.15210 (PMC10864115; doi:10.1111/1759-7714.15210)
Supplement: Supplementary file 1 — Supplementary Table S1. Efficacy of osimertinib. Supplement Table S2. Adverse events. [file TCA-15-402-s001.docx]

**Supplement Table 1. Efficacy of osimertinib**

**CR: Complete response, PR: Partial response, SD: Stable disease, NE: Not evaluate, ORR: Overall response rate, DOR: Disease control rate, PFS: progression-free rate, OS: Overall survival, CI: confidence interval.**

|  | EGFR-TKI naïve | | Acquired T790M | |
| --- | --- | --- | --- | --- |
|  | With MPE | Without MPE | With MPE | Without MPE |
|  | (N=45) | (N=68) | (N=39) | (N=77) |
| Response |  |  |  |  |
| CR | 0 | 0 | 0 | 0 |
| PR | 18 | 34 | 19 | 44 |
| SD | 21 | 28 | 16 | 25 |
| PD | 2 | 5 | 2 | 5 |
| NE | 4 | 1 | 2 | 3 |
| ORR | 40.0 % | 50.0 % | 48.7 % | 59.7 % |
| DOR | 86.6 % | 91.2 % | 89.7 % | 89.6 % |
| Median PFS (months) (95%CI) | 14.8 (12.8-19.8) | 19.8 (9.8-29.8) | 12.3 (9.3-16.3) | 13.1 (10.3-16.4) |
| Median OS (months) (95%CI) | 32.0 (29.4-43.0) | 42.0 (32.0-52.7) | 23.2 (13.9-30.6) | 24.7 (19.1-32.0) |

**Supplement Table 2. Adverse Events**

**ILD: Interstitial lung disease.**

|  | All patients | EGFR-TKI naïve | | Acquired T790M | |
| --- | --- | --- | --- | --- | --- |
|  |  | With MPE | Without MPE | With MPE | Without MPE |
|  | (N-229) | (N=45) | (N=68) | (N=39) | (N=77) |
| TRAE (Any Grade / > Gradae3) |  |  |  |  |  |
| Rash | 144 / 14 | 30 / 4 | 42 / 2 | 23 / 5 | 49 / 3 |
| Paronychia | 80 / 0 | 13 / 0 | 23 / 0 | 16 / 0 | 28 / 0 |
| ILD | 20 / 4 | 2 / 1 | 7 / 1 | 4 / 2 | 7 / 1 |
| Diarrhea | 15 / 1 | 5 / 0 | 5 / 1 | 3 / 0 | 2 / 0 |
| Neutropenia | 12 / 5 | 4 / 0 | 3 / 1 | 0 / 0 | 5 / 4 |
| Liver disfunction | 10 / 5 | 3 / 0 | 2 / 1 | 2 / 2 | 3 / 2 |
| Mucositis oral | 8 / 0 | 2 / 0 | 4 / 0 | 0 / 0 | 2 / 0 |
| Anorexia | 4 / 0 | 0 / 0 | 0 / 0 | 1 / 0 | 3 / 0 |
| Fever | 3 / 0 | 1 / 0 | 2 / 0 | 0 / 0 | 0 / 0 |
| CK elevation | 3 / 0 | 1 / 0 | 1 / 0 | 0 / 0 | 1 / 0 |
| Cr elevation | 2 / 0 | 1 / 0 | 0 / 0 | 0 / 0 | 1 / 0 |
| Others | 5 / 0 | 2 / 0 | 1 / 0 | 1 / 0 | 1 / 0 |
| Dose reduction | 23 | 5 | 6 | 3 | 9 |
| Treatment discontinue | 20 | 4 | 8 | 3 | 5 |
